# Supplementary material for: Frailty and depression predict instrumental activities of daily living in older adults: A population-based longitudinal study using the CARE75+ cohort
Source: PLoS One. 2020 Dec 15;15(12):e0243972. doi: 10.1371/journal.pone.0243972 (PMC7737980; doi:10.1371/journal.pone.0243972)
Supplement: Attachment — Submitted filename: Review PONE-D-20-09338.docx [file pone.0243972.s002.docx]

**Review PONE-20-09338 “Frailty and depression predict instrumental activities of daily living in older adults: a population-based longitudinal study using the CARE75+ cohort**

The authors used a subsample of participants in the CARE75+ cohort to examine the association between depression, frailty and instrumental activities of daily living (IADL) from baseline to 12 months of follow-up. The analytic approach is a linear mixed model with random intercept. The presentation and presentation of results can be improved. Answers to the following questions can improve a reader’s understanding of the validity of the results and conclusions.

1). Based on the flow chart, 106 participants were excluded from the study due to “delay between participant consent and baseline data being uploaded.” Were their baseline data available? If not, they probably would have been counted as “lost to study entry.” So my guess is their data were available. Then, what is the justification for this exclusion?

2). There was substantial amount of attrition (39% at 6 months and 60% at 6 months). It is known that linear mixed effects models are consistent under the assumption of missing at random. However, given this cohort’s age, it is unlikely the attrition was at random. The authors were not clear how many died, transitioned to a care home and was not followed, and was lost to follow-up due to other reasons. When attrition is not at random, the mixed effects model estimates are biased.

3). After estimating a linear mixed model, the authors presented marginal effects in Figure 2. Given that the analysis was done in Stata, I assume the authors used the margins statement after the linear mixed model for the marginal effects. However, the margins command after a mixed model in Stata only calculates the fixed effects part of the model, thus is not a real marginalization. Granted that if the missing at random assumption holds, the fixed part of the mixed model is equivalent to the population-averaged effects; however, given 2) this is unlikely to be true. The authors could take a look at the article by Rouanet et al. (2019) “Interpretation of mixed models and marginal models with cohort attrition due to death and drop-out” in Statistical Methods in Medical Research.

4). The authors speculated that “the absence of an effect in those with severe frailty stem from an underpowered analysis owing to smaller numbers.” However, the authors did not present the number of people with depression and severe frailty in each time period. A tabulate of sample size over time will be helpful.
