# Supplementary material for: Frailty and depression predict instrumental activities of daily living in older adults: A population-based longitudinal study using the CARE75+ cohort
Source: PLoS One. 2020 Dec 15;15(12):e0243972. doi: 10.1371/journal.pone.0243972 (PMC7737980; doi:10.1371/journal.pone.0243972)
Supplement: Attachment — Submitted filename: Responses to the reviewers_v2.docx [file pone.0243972.s003.docx]

Responses to the reviewers

| **Reviewer one comments** | **Author responses** |
| --- | --- |
| The manuscript is interesting. However, I have some concerns about the number of patients. What about the sample size and the power of the study? What about the comorbidities such as heart failure? Please see and discuss Liguori I et al. Depression and chronic heart failure in the elderly: an intriguing relationship. J Geriatr Cardiol. 2018 Jun;15(6):451-459. | This study is based on analysis of an observational cohort and used all available data on participants. While the cohort size was not based on the current research question, a sample of 553 with up to three observation time points is considered sufficient to detect significant associations if they exist.  We agree that comorbidities such as cardiac conditions are commonly associated with depression and activities of daily living. We used the Katz index of comorbidities and included this as a covariate in our regression analysis. This index captures data on cardiac and other long-term physical and mental health conditions. |
| **Reviewer two comments** | **Author responses** |
| Based on the flow chart, 106 participants were excluded from the study due to “delay between participant consent and baseline data being uploaded.” Were their baseline data available? If not, they probably would have been counted as “lost to study entry.” So my guess is their data were available. Then, what is the justification for this exclusion? | We have amended to flow chart to reflect the fact that these 106 participants had been recruited and consented into the study but their baseline data were unavailable at the time of the data extract for this study.  This is due to the fact that the consent procedure for CARE75+ is quite complex, with multiple and time consuming study components. The consent is *often* carried out on a separate visit to the baseline assessment visit. As such some people may have provided consent to take part and then not been seen for some time to undertake their assessments (holidays and ill-health can delay this). Furthermore, assessments are sometimes undertaken over multiple visits (for example if the person has frailty and gets tired). Finally, as the study involves collecting data from the GP practices (medication and comorbidities), there can be a delay in researchers accessing and inputting that data onto the database. Hence there is a discrepancy between the number of those that consented to take part and the available data used in this study. |
| There was substantial amount of attrition (39% at 6 months and 60% at 6 months). It is known that linear mixed effects models are consistent under the assumption of missing at random. However, given this cohort’s age, it is unlikely the attrition was at random. The authors were not clear how many died, transitioned to a care home and was not followed, and was lost to follow-up due to other reasons. When attrition is not at random, the mixed effects model estimates are biased. | Thank you for raising this point. The study recorded a total of 14 deaths; half of these participants, i.e. 7/14, completed all three waves of the study (i.e. they died after the 12-month follow-up period). As a result, deaths accounted for only 7 cases of missing data during the study follow-up. The study did not collect data on possible transition to a care home.  Based on the reviewer’s comment, we investigated the association between loss to follow-up and age of the participant as well as their frailty level (based on EFI score) and depression level (based on GDS-15). For this: (a) we conducted a logistic regression with binary variable representing loss to follow-up at 6 or 12-months (coded 1) vs not (0) using age and baseline frailty and depression as predictors; and (b) the same logistic regression model as above but using age and lagged values of depression level and frailty score in the previous observation timepoint (e.g. the 6-month frailty level was a predictor for loss to follow-up at 12-month timepoint). We found that none of the variables in these analyses had significant associations with missingness/loss to follow-up. The results are presented below. We have also added text on page X to explain this. |
| After estimating a linear mixed model, the authors presented marginal effects in Figure 2. Given that the analysis was done in Stata, I assume the authors used the margins statement after the linear mixed model for the marginal effects. However, the margins command after a mixed model in Stata only calculates the fixed effects part of the model, thus is not a real marginalization. Granted that if the missing at random assumption holds, the fixed part of the mixed model is equivalent to the population-averaged effects; however, given 2) this is unlikely to be true. The authors could take a look at the article by Rouanet et al. (2019) “Interpretation of mixed models and marginal models with cohort attrition due to death and drop-out” in Statistical Methods in Medical Research. | Thank you for sharing the reference (Rouanet et al. 2019) which shows that mixed models may be problematic when attrition is due to death. However, as per the previous response, there were only 7 deaths before the 12-month time point. Therefore, we do not believe that attrition due to death would have had a significant impact on our analyses. We have now included text on page X to clarify that the marginal effects represent population-averaged effects. |
| The authors speculated that “the absence of an effect in those with severe frailty stem from an underpowered analysis owing to smaller numbers.” However, the authors did not present the number of people with depression and severe frailty in each time period. A tabulate of sample size over time will be helpful | We present a table of depression and frailty index below. Based on further discussion with clinicians,  another plausible explanation is that severe frailty in itself is so debilitating for activities of daily living that any moderating effect at that level is non-significant. We have added a line in the paper to this effect. |
| I have some concerns about adjusting confounders: given that cognitive impairment is an independent factor for ADL and IADL loss and that depression and dementia are often overlapping in the elderly population, has cognitive impairment been evaluated in the sample? Furthermore, GDS is not a validated tool for the evaluation of depression in patients with cognitive impairment. Indeed I think that some clarifications are necessary about effects of depression symptoms on comorbidities, please see and discuss “Depressive symptoms predict mortality in elderly subjects with chronic heart failure. Testa G, Cacciatore F, Galizia G, Della-Morte D, Mazzella F, Gargiulo G, Langellotto A, Raucci C, Ferrara N, Rengo F, Abete P. Eur J Clin Invest. 2011 Dec;41(12):1310-7”. | The reviewer raises an important point about the overlap. We do not have a measure of cognitive impairment and we acknowledge this as a limitation in the discussion section. We have now included in the discussion a statement that acknowledges that there remains a debate about the extent to which the GDS is validated in people with cognitive impairment. This debate is further elaborated on by acknowledging the findings from the study by Testa et al (2011) which showed that the GDS had proven predictive validity in older adult populations with CHF, disability, and cognitive impairments. |
