# Supplementary material for: Frailty and depression predict instrumental activities of daily living in older adults: A population-based longitudinal study using the CARE75+ cohort
Source: PLoS One. 2020 Dec 15;15(12):e0243972. doi: 10.1371/journal.pone.0243972 (PMC7737980; doi:10.1371/journal.pone.0243972)
Supplement: Attachment — Submitted filename: Responses to the reviewers_revision_v3.docx [file pone.0243972.s004.docx]

Responses to the reviewers

| **Reviewer two comments** | **Author responses** |
| --- | --- |
| The authors responded to my second question by saying “The study recorded a total of 14 deaths; half of these participants, i.e. 7/14, completed all three waves of the study (i.e. they died after the 12-month follow-up period).” In the main text this information should be given. In the follow chart and in the main text, the reason for loss to follow up (not death) should be given, at six months 214 (7 died) patients were not included ; and at 12 months 331 (7 died) patients were not included; what are the reason for the exclusions? | We have now included the detail about the number of deaths recorded to the introductory paragraph of the results.  We have explained in the limitations section of the discussion that the omissions from 6-month and 12-month follow-up owe to the fact that 214 and 331 participants respectively had not reached these data collection time points at the time of the data extraction and analysis. In this sense they do not qualify as losses to follow-up but are a product of lag between data collection, data extract, and data analysis.  We apologise that this was unclear in the original submission and in our previous response. |
| The model for 12-month loss to follow up should not be among only those patients with non-missing 6-month data, it should be among all 553 patients. The authors should show in their response letter the logistic regression for loss to follow up at 6 months with age and baseline frailty and depression as predictors, although I do not believe this necessarily mean anything because we still don’t know if other variables are related to loss to follow up. | As clarified above, missing outcome data at 6 and 12 month timepoints is primarily due to lag in data accrual. However, we still evaluated if unavailable 6-month follow-up data was associated with age, baseline frailty and depression. However, we found that none of these were significant predictors (see table A1 below). |
| Descriptive statistics of all variables in Table 1 should be given for those with and those without loss to follow up, one table for 6-month; one table for 12-month. We can from the descriptive statistics get a glimpse of how different the two groups are. | Table 1 as is convention describes the characteristics of the recruited cohort at baseline. This includes descriptive date for NEADL and depression at baseline. Given the clarification above, we do not see the utility of providing descriptive statistics for 6-months and 12-months, particularly as many of the descriptive variables are non-modifiable and unlikely to change. However, we have provided summaries of depression status by frailty status at 6-month and 12-months in Table 2. |
| The authors added Table 2 (cross-tabulation of depression and frailty at baseline, 6 months and 12 months) in response to my question about their speculation that “the absence of an effect in those with severe frailty stem from an underpowered analysis owing to smaller numbers”, we can see that there are only 2 patients with depression and no frailty in 12 months. This fact makes the models with interaction between depression and frailty very questionable. No descriptive data were given for the outcome NEAL at baseline, 6 months or 12 months. One cannot get a sense of how big the effects (coefficients) are relative to the distribution of the outcome. | Table 1 includes the baseline NEADL score, and we have now included both 6-month and 12-month scores in the results section. Also, we previously acknowledged in the discussion section that the small number of participants with depressive symptoms, particularly those with severe frailty, have likely resulted in large uncertainty in estimates of the interaction analysis. |

Table A1: Logistic regression for unavailable 6-month follow-up data (dependent variable), with baseline predictors

| **Dependent var (0/1): unavailable follow-up data at 6-month** | **Odds Ratio** | **SE** | **z** | **P>z** | **[95% Conf.** | **Interval]** |
| --- | --- | --- | --- | --- | --- | --- |
| Age (in years) | 1.021 | 0.023 | 0.920 | 0.356 | 0.977 | 1.066 |
| EFI: reference (reference: fit) |  |  |  |  |  |  |
| EFI: mild frailty | 0.772 | 0.220 | -0.910 | 0.365 | 0.441 | 1.351 |
| EFI: moderate frailty | 0.658 | 0.199 | -1.380 | 0.167 | 0.364 | 1.191 |
| EFI: severe frailty | 0.858 | 0.350 | -0.370 | 0.708 | 0.386 | 1.909 |
|  |  |  |  |  |  |  |
| Depressed (based on GDS-15) | 0.869 | 0.261 | -0.470 | 0.641 | 0.482 | 1.567 |
| Constant | 0.748 | 0.170 | -1.280 | 0.201 | 0.479 | 1.168 |
